# Increasing nontuberculous mycobacteria reporting rates and species diversity identified in clinical laboratory reports

**DOI:** 10.1186/s12879-018-3043-7

**Published:** 2018-04-10

**Authors:** Maura J. Donohue

**Affiliations:** United States Environmental Protection Agency, 26 W. Martin Luther King Dr. Mail Stop 587, Cincinnati, Ohio 45268 USA

**Keywords:** Nontuberculous mycobacteria (NTM), Clinical laboratory reports, Species, Epidemiology, Isolation rates, Report rates, Seasonal, United States

## Abstract

**Background:**

Nontuberculous Mycobacteria (NTM) are environmental microorganisms that can affect human health. A 2009–2010 occurrence survey of NTM in potable tap water samples indicated an increased recovery rate for many clinically significant species such as *M. avium* (30%) and *M. abscessus* (12%). To determine if these trends by species were mirrored in human infections, isolation rates of NTM species identified in clinical laboratory reports from four states were evaluated.

**Method:**

Clinical laboratory reports from the Mississippi, Missouri, Ohio, and Wisconsin Health Departments were used to investigate the species of NTM isolated from human specimens in 2014. The NTM positive specimen reports were tabulated for each species and complex/group. The number of reports by month were used to investigate seasonal trends. The 2014 isolation rates were compared to historic values to examine longitudinal trends.

**Results:**

The positive rate of NTM specimens increased from 8.2 per 100,000 persons in 1994 to 16 per 100,000 persons in 2014 (or 13.3 per 100,000 after excluding *Mycobacterium gordonae*). Changes in NTM diversity were observed in complex/groups known to be clinically significant. Between 1994 and 2014 the rate implicating *M. abscesses-chelonae* group and *M. avium* complex increased by 322 and 149%, respectively.

**Conclusions:**

Based on public health data supplied by the four State’s Health Departments and the 2014 U.S. population, 50,976 positive NTM specimen reports per year were projected for the nation; serving as an indicator for the national potential disease burden that year.

**Electronic supplementary material:**

The online version of this article (10.1186/s12879-018-3043-7) contains supplementary material, which is available to authorized users.

## Background

Data from recent studies demonstrate an increasing prevalence of Nontuberculous Mycobacteria (NTM) infections in the United States [1–3]. NTM are of environmental origin and can impact a wide variety of tissues and body fluids causing both respiratory (chronic bronchopulmonary disease) illnesses and dermal infections [4]. NTM infections may also compound the respiratory ailments of individuals with Cystic Fibrosis [5], Chronic Obstructive Pulmonary Disease (COPD) [6, 7] and Acquired Immunodeficiency Syndrome (AIDS) [8, 9].

NTM’s natural habitats are aquatic and soil environments [10]. Since the majority (77.4% [11] to 91.5% [12]) of NTMs are isolates from pulmonary specimens, exposure likely occurs through the aerosolization or aspiration of water and or soil particulates. NTM infections or illnesses are not generally communicable, although there are a few documented cases of person-to-person transmission [13, 14]. Additionally, highly populated areas have a higher NTM infection rate and positive specimen counts than other regions of the U.S. [15, 16].

NTM is a broad classification term that is applied to a group of approximately 186 currently recognized unique mycobacterium species [17]. The majority do not have an impact on human health. Thus, it is important to identify the species causing an infection in cases where symptoms are sufficient to support specimen collection. Knowledge of the NTM species identity will guide the therapeutic treatment prescribed by the physician and provide clues relative to exposure source and route.

This study catalogued the NTM species in human-specimen clinical reports from four States (Mississippi, Missouri, Ohio, and Wisconsin) during the 2014 calendar year with the goal of identifying the species with the most frequent impact on human health. The dates for the case report submissions were examined to determine if there was any indication of seasonality. In addition, the 2014 findings were compared to those from an earlier 1994 Centers for Disease Control and Prevention (CDC) report of NTM infections (19) to evaluate if prevalence had increased over the last 20 years.

## Methods

### Study design

Four states (Mississippi, Missouri, Ohio, and Wisconsin) that required NTM positive specimen results to be submitted to their respective state health departments were asked if they would provide the following data from their disease surveillance network: the number of positive NTM reports by species and the number of positive NTM reports by month for the 2014 calendar year. The respective health departments agreed to participate and provided the requested information to the U.S. EPA. Table 1 summarizes the information received from the four states. No personal identifiers were shared with U.S. EPA, therefore maintaining the privacy for the individuals associated with all report records. This study was determined to be exempt from Institution Review Board review by U.S. EPA.

**Table 1:** Health departments data sources and NTM identification method for the year 2014

State	Data source	Year	NTM identification method	NTM laboratory report	Report rate^a^
Mississippi	Mississippi’s public health				
	laboratories + accredited	2014	HPLC and DNA probes	529	17.6
	laboratories (hospitals and/01				
	commercial laboratories)				
Missouri	Missouri’s public health		HPLC and DNA probes		
	laboratories + few hospitals	2014		879	14.5
	+ commercial laboratories				
Ohio	Ohio’s public health laboratories + hospitals + commercial laboratories	2014	HPLC, DNA probes, sequencing of the *hsp65* and *rpoB* gene and biochemical test	1379	11.8
Wisconsin			HPLC and DNA		
		2014	probes and inconclusive isolates are sequenced.	1413	24.5
Total				4200	16.0

^a^ Rate per 100,000 persons

### Data

Four health departments provided U.S. EPA with the number of NTM reports by species for the 2014 calendar year. The methods used to isolate NTMs were either through culture [18] or the Becton Dixon BACTEC™ Mycobacteria Growth Indicator Tube (MGIT™) [19]. All four states largely used mycolic acid analysis and DNA probes (Hologic, Marlborough, MA) for species identification. Some laboratories sequenced an isolate’s *hsp65* or *rpoB* gene for NTM identification (Table 1).

Cases implicating all non-NTM isolates, as well as, isolates of *M. bovis* and *M. marinum* were removed from the data set for this analysis. Cattle and fish, not water, are the sources of infection for *M. bovis* and *M. marinum* species; thus, they were not considered in this evaluation*.*

### Analysis

Report rates for NTMs were calculated for each state by dividing the number of positive NTM case reports by the July 1, 2014 state population. Calculation of these rates used 2014 U.S. population estimates for each state as follows (Table 1): Mississippi: 2,993,443; Missouri: 6,063,827; Ohio: 11,597,998; Wisconsin: 5,759,432 and U.S.: 318,563,456. The percentage of the total was calculated for each species or group identified by each state using Microsoft Excel.

CDC does not routinely collect NTM specimen reports. Periodically, over the past 40 years, CDC has published a few papers and reports that examined the epidemiology of NTMs in the U.S. These papers examined NTM prevalence by the number of laboratory reports generated by each state [20–22]. The 1994 historical data for each of the four states was extracted from the Butler and Crawford, 1999 document. This document contains the number of reports and report rate by species for each state in the U.S for the years 1993–1996. In the 1990’s, laboratories were using either culture bases methods [18] or BD MGIT™ systems [19] for bacterial enrichment and DNA/RNA probes (Hologic, Marlborough, MA) and or mycolic acid analysis for identification [23]. In 2014, the majority of laboratories were still using these culture and identification methods. Thus, supporting a comparison between the two-time periods 1994 and 2014. Chi-square and Fisher Exact tests for significant differences between the two data sets were determined using SigmaPlot 13.0.

### Species grouping

Many of the NTM species have similar characteristics and have been placed into groups of two to seven species (Table 2) with some species being ungrouped. The species identified by the states were summed by their group for this analysis to facilitate a comparison between the historical 1994 data and the 2014 data. For example, 1541 samples identified as M. *avium* complex (MAC) positive specimens were combined with 705 reports identified as *M. avium* species and 6 reports identified as *M. intracellulare* species to give a total of 2252 reports for the MAC group. Table 2 lists the NTM species reports that were combined to represent each NTM group/complex/clad unit for the comparison.

**Table 2:** NTMs identified from human specimens in Mississippi, Missouri, Ohio, and Wisconsin, 2014

Identified to Genus	Complex/Group Totals	Identified to NTM Complex/Group	Identified to NTM Species	Count	Percentage	Rate^a^
NTM				132	3.14	0.50
	MAC complex (Total)			2252	53.62	8.53
		MAC complex		1541	36.69	5.83
			*M. avium*	705	16.79	2.67
			*M. intracellulare*	6	0.14	0.02
	MAIS Complex (Total)			20	0.48	0.08
		MAIS Complex		17	0.40	0.06
			*M. scrofulaceum*	3	0.07	0.01
	M. *chelonae-abscessus* Group (Total)			440	10.48	1.67
		*M. chelonae-abscessus* Group		122	2.90	0.46
			*M. abscessus*	62	1.48	0.23
			*M. bolletii*	5	0.12	0.51
			*M. chelonae*	135	3.21	0.00
			*M. franklinii*	1	0.02	0.02
			*M. immunogenum*	4	0.10	
			*M. massiliense*	2	0.05	
			*M. mucogenicum*	104	248	0.39
			*M. phocaicum*	5	0.12	0.02
	*M. fortuitum* Group (Total)			343	8.17	1.30
		*M. fortuitum* Group		143	3.40	0.54
			*M. conceptionense*	2	0.05	0.01
			*M. fortuitum*	110	2.62	0.42
			*M. houstonense*	1	0.02	0.00
			*M. neworleansense*	2	0.05	0.01
			*N. peregrinum*	76	1.81	0.29
			*M. porcinum*	5	0.12	0.02
			*M. septicum*	4	0.10	0.02
	*M. gordonae* (Total)		*M. gordonae*	689	16.40	2.61
	*M. xenopi* (Total)		*M. xenopi*	54	1.29	0.20
	*M. kansasii* Clade (Total)			100	2.38	0.38
			*M. gastri*	1	0.02	0.00
			*M. kansasii*	99	2.36	0.37
	*M. haemophilum* (Total)			7	0.17	0.03
			*M. haemophilum*	2	0.05	0.01
			*M. malmoense*	5	0.12	0.02
	*M. terrae* Complex (Total)			60	1.43	0.23
			*M. arupense*	21	0.50	0.08
			*M. kumamotonense*	1	0.02	0.00
			*M. nonchromogenicum*	10	0.24	0.04
			*M. terrae*	28	0.67	0.11
	*M. smegmatis* (Total)			17	0.40	0.06
			*M. goodii*	7	0.17	0.03
			*M. mageritense*	4	0.10	0.02
			*M. smegmatis*	4	0.10	0.02
			*M. wolinskyi*	2	0.05	0.01
	*M. simiae* Complex (Total)			45	1.07	0.17
			*M. interjectum*	2	0.05	0.01
			*M. kubicae*	2	0.07	0.01
			*M. lentiflavum*	15	0.36	0.06
			*M. parascrofulaceum*	2	0.05	0.01
			*M. simiae*	22	0.52	0.08
			*M. triplex*	1	0.02	0.00
	Ungrouped NTM (Total)			41	0.98	0.16
			*M. algericum*	1	0.02	0.00
			*M. aurum*	1	0.02	0.00
			*M. bacteremicum*	1	0.02	0.00
			*M. branderi*	1	0.02	0.00
			*M. celatum*	1	0.02	0.00
			*M. cosmeticum*	4	0.10	0.02
			*M. frederiksbergense*	1	0.02	0.00
			*M. iranicum*	1	0.02	0.00
			*M. nebraskense*	2	0.05	0.01
			*M. neoaurum*	6	0.14	0.02
			*M. paraffinicum*	9	0.21	0.03
			*M. pulveris*	1	0.02	0.00
			*M. sphagni*	1	0.02	0.00
			*M. szulgai*	10	0.24	0.04
			*M. vaccae*	1	0.02	0.00

^a^Rate per 100,000 persons

### Monthly reports

The Mississippi, Missouri, and Ohio Health Departments provided the U.S. EPA with the number of NTM specimen reports by month for the year 2014. Mississippi data are publicly available at the Monthly Reportable Disease Statistics website: http://msdh.ms.gov/msdhsite/_static/14,0,261.html. The Wisconsin Health Department was unable to supply their data in this format; thus, there is no evaluation of cases per month for the state of Wisconsin. The monthly report data were used to determine if report frequency exhibited any seasonal trends.

## Results

### NTM species in laboratory reports

The Mississippi, Missouri, Ohio, and Wisconsin disease network had 4200 NTM positive specimen reports submitted in 2014. The overall NTM prevalence rate was 16.0 reports per 100,000 persons (or 13.3 per 100,000 excluding *Mycobacterium gordonae*). This rate is much higher than that reported by the CDC in 1994. Based on the 2014 data, a national estimate of 50,976 positive NTM specimen reports are projected during that year.

The distributions by state for the total NTM positive specimens are as follows: Wisconsin (33.6%), Ohio (32.8%), Missouri (20.9%) and Mississippi (12.6%) (Additional file 1: Table S1-S4). The complex/group assignment to which an isolate was speciated varied across the states. Three percent of the reports identified the isolates only to the genus-level. Forty-three percent of the reports assigned the isolates only to a specific complex/group, and 54% of reports provided the species name (Table 2). In all, 52 species were identified by name in the state reports. Isolates classified only by their complex/group designation primarily belonged to the *M. avium* complex (MAC), *M. chelonae-abscessus* or *M. fortuitum* groups. Table 2 provides the data for all the species identified and ascribes each species to its designated complex or group. The five most common species isolated from human specimens were *M. avium* 705/4200 (17%), *M. gordonae* 688/4200 (16%), *M. chelonae* 135/4200 (3.2%), *M. fortuitum* 110/4200 (2.6%), and *M. mucogenicum* 104/4200 (2.5%) (Table 2). These species accounted for 41.4% of the total demonstrating the broad range of species identified among the samples collected for analysis.

### NTM increased reporting frequency

**Fig. 1:**
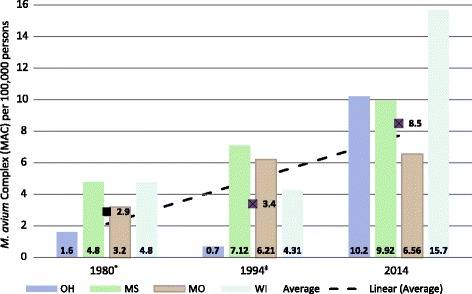
Numbers, rates, and percent change of NTM reports by species, 1994 and 2014 comparison. See Table 2 for clarity on which NTM species were considered in each complex or group. *2014: *M. chelonae-abscessus* Group plus. *M. mucogenicum-phocaicum* report numbers were combined, ‡ NS = Not significant, § per 100,000 persons: 1994 population 23,795,000 (as reported by Butler and Crawford, 1999); 2014 population 26,414,700

NTM positive specimen reports have increased between 1994 and 2014 from 1950 to 2400, equivalent to an increase of 8.2 per 100,000 persons per year to 16.0 per 100,000 persons per year. The three complexes/groups that had statistically significant increases in the frequency of detections were the: *M. chelonae*-*abscessus* group, *M. fortuitum* group and *M. avium* complex. The percentage increase of these clinically important species, as established by American Thoracic Society and Infectious Disease Society of America, (ATS/IDSA) [4] between 1994 and 2014 is significant: 322% (94 to 440 reports), 194% (105 to 343 reports), and 149% (816 to 2252 reports), respectively (Fig. 1). On the other hand, Fig. 1 illustrates a decline in the number of positive specimen reports for other species: *M. xenopi (− 15%)*, *M. kansasii (− 30%), M. terrae (− 40%)*, *M. scrofulaceum (− 55%)* and *M. flavescens* (none detected).

### Longitudinal analysis for the MAC species

**Fig. 2:**
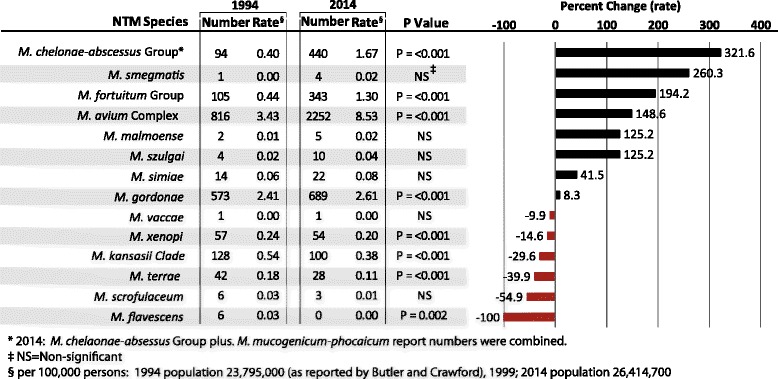
Longitudinal analysis (1980–2014) of *M. avium* complex report rate per 100,000 persons for Mississippi, Missouri, Ohio, Wisconsin, and combined. * Butler and Crawford, 1999: 1994 rates

Figure 2 illustrates the report rate of *M. avium per* 100,000 persons over time for the four states. In 2014, the combined report rate of *M. avium* for Mississippi, Missouri, Ohio, and Wisconsin was 8.5 per 100,000 persons (Fig. 2). This is 2.5 times more reports than the numbers reported in 1994 [20]. The species detections for Ohio and Wisconsin increased more than five-fold suggesting a comparable increase in the respiratory infections disease burden.

### NTMs reports by month

**Fig. 3:**
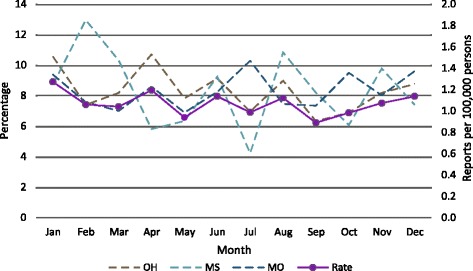
NTM reports by state by month

When the 2014 positive specimens per month for each state were plotted (Fig. 3) there was no evidence for a seasonal pattern to the potential disease burden. The peak month for each state differed: February for Mississippi, April for Ohio, and July for Missouri. Data from Wisconsin were not segregated by month.

## Discussion

Case reports are the bedrock for disease surveillance in the U.S. Unfortunately, CDC’s National Notifiable Disease Surveillance System (NNDSS) does not routinely collect data on NTM positive laboratory reports or disease cases on a national basis. In recent years, state health departments are allowing other public and private laboratories to submit reports into their disease surveillance systems due to the increase capability of private and public labs to identify NTMs in human specimens and improvements in electronic reporting. The state NTM data reports capture information that reflects potential respiratory infections among both the young and elderly, as well as, the poor and the uninsured population groups often underrepresented in studies of disease burden based on Medicare part B and/or health care insurance records. However, there are limitations to these state data. The NTM reports are not generally scrutinized for accuracy or completeness during data entry. The procedures for isolating the NTM species from the specimen could yield false positives due to laboratory contamination. In addition, more than one report could belong to the same individual if more than one specimen was collected during the course of a single disease episode in order to evaluate treatment efficacy. Thus, the NTM specimen reports can overestimate the burden of disease. Yet, the report rate of 13.3 per 100,000 is similar to 13.7 per 100,000 persons (North Carolina) [12] and 17.2 reports per 100,000 persons (Oregon) [11] other estimates of NTM prevalence in the U.S. Additionally, the estimate of 50,976 NTM reports in the U.S. per annum derived by using the state NTM reports is of the same order of magnitude as the estimated 86,244 cases identified using Medicare part B records [24]. Multiple lines of evidence demonstrate an increase in NTM prevalence [1–3] and report rates [12], strengthening the conclusion that NTM infections in the U.S. have increased. These lines of evidence also elevate the concern for NTM-related diseases in the U.S., identifying a need to search for controllable sources of exposure that will guide risk mitigation measures [25].

In this study, fifty-two NTM species were identified from human specimens. Twenty eight percent (52/186) of recognized NTM species are represented in the specimen reports. The four most frequently identified NTM groups or species that were common across the four states were: MAC, *M. fortuitum*, *M. gordonae*, and *M. chelonae-abscessus* groups. This observation was supported by data from other regions and states within the U.S. [11, 12, 26].

The increase in the number of NTM positive reports is not solely due to population growth. From 1994 to 2014, the population in these four states increased by 2.1 million people. Using the CDC 1994 report rate of 8.2 per 100,000 persons [20], this population increase should have only added 172 reports. Instead the number of reports for the four states is 4200 positive reports. This number was greater than 2000 more reports than predicted. The reason for this increase is probably multifactorial. One important factor that likely led to the larger case count is the increase in electronic reporting by laboratories who perform sample analyses to state databases. Established common portals for data entry can be used by state laboratories, large research hospitals, as well as, commercial laboratories to facilitate improved record keeping. However, despite the impact of technology on better reporting, the increase in the number of reports is substantial enough to support the conclusion that more individuals are experiencing NTM related episodes impacting their health now than they did two decades ago.

There was an increase in the MAC positive reports from 3.4 per 100,000 in 1994 to 8.5 per 100,000 in 2014 (Fig. 2). Numerous factors could have contributed to the increase in prevalence including: increased medical awareness of NTM related infections/diseases, improvement of laboratory identification techniques, increases in exposure modalities and human activities associated with water and/or soils. The increased MAC detection rates for the four states suggest a need for improved risk communication efforts between the public health sector and populations at risk for NTM infections. The NTM family of microorganisms and their contribution to respiratory illnesses are seldom topics for the popular press and media. There is a need for a better communications strategy targeting the general population on both the signs and symptoms of NTM infections and measures that will help minimize risk.

The shifts in detection frequency for either an NTM group or species should be considered in any assessment of population risk. Not all NTM species have the same symptoms nor impact on human health. Species within the *M. chelonae*-*abscessus* and *M. fortuitum* groups are those commonly identified in “outbreak” investigations [27–29]. These outbreaks can include dermal infections of either the skin or soft tissue and pulmonary problems. Dermal signs and symptoms reported were immediate post contact rash onset, septic arthritis, or cutaneous infections of the skin [27–29]. The source of the infection is typically found to be a non-potable or potable water source that has been contaminated with NTMs. Species in the MAC grouping have been the most clinically significant because they cause the majority of NTM related illnesses and diseases [3, 30].

The increases in a state’s population alone do not account for the increased number of cases reported by the state health departments. However, changes within the population can contribute to the increase. For example, a state population could contain more elderly individuals with co-morbidity factors such as COPD or people suffering from suppression of their immune response than in the past. Changes in routes of exposure are also important. MAC infections are usually respiratory, but not communicable [4]. Therefore, changes in bathroom construction that promote showering over bathing (especially for the elderly) increases the likelihood for inhalation exposure and infection when potable water is the source of transmission. A recent survey of NTM presence in tap water found 78% (202/258) of the samples collected were positive for NTM [31]. Since inhalation is believed to be the main route of exposure [4], the increased use of shower water aerators could also be an important feature associated with an increase in population exposure [32]. Recently, several publications [33] have outlined measures, such as, cleaning showerheads and removing aerators as protective measures for avoiding exposure to NTMs [34, 35].

There is no seasonal pattern to NTM reports. The lack of seasonality suggests exposure routes for illness and or disease occur year-round and may not be influenced by seasonal changes in activities of the at-risk population. This is probably due to the fact that pulmonary NTM lung infections and/or diseases are not acute illnesses. Common signs and symptoms of the respiratory problems are shortness of breath and fatigue [4]. These vague symptoms may not be addressed in a timely manner, lengthening the time between illness onset and consultation with a physician because of symptom persistence. This delay could potentially dampen the opportunity to link a positive specimen as a result of physician consultation to the time the symptoms were first noted. As a result, a potential NTM report is filed with the state long past the time of infection acquisition.

## Conclusion

Knowledge of the NTM species most frequently associated with adverse human health consequences will assist epidemiological investigators to identify likely sources and modes of exposure associated with these types of infections. By identifying the sources of the NTM infections, strategies can be implemented to control for the occurrence of NTMs and/or procedures can be adopted to mitigate exposure routes associated with the risks. NTMs and their related disease manifestations result in a significant, medical, human health burden, especially among the elderly [2, 15]. As the U.S. population ages, the public health burden from NTM associated disorders is expected to increase. Thus, it is important to conduct research to identify the major exposure routes and environmental sources of dissemination to identify and implement practices that will limit human exposures to NTM infections.

### Disclaimer

The United States Environmental Protection Agency through its Office of Research and Development funded and managed the research described here. It has been subjected to Agency’s administrative review and approved for publication. The views expressed in this paper are those of the authors and do not necessarily reflect the views or policies of the United States Environmental Protection Agency.

## Additional file

Additional file 1: **Table S1.** Nontuberculous mycobacteria reported by Missouri, 2014. **Table S2.** Nontuberculous mycobacteria reported by Mississippi, 2014; **Table S3.** Nontuberculous mycobacteria reported by Ohio, 2014. **Table S4.** Nontuberculous mycobacteria reported by Wisconsin, 2014. Provisional counts of NTM reports by State and NTM Species. (DOCX 26 kb)

## Abbreviations

AIDS: Acquired immunodeficiency syndrome
ATS/IDSA: American thoracic society and infectious disease society of America
CDC: Center for disease control and prevention
COPD: Chronic obstructive pulmonary disease
MAC: *M. avium* complex
NNDSS: National notifiable disease surveillance system
NTM: Nontuberculous mycobacteria
U.S. EPA: United States environmental protection agency
U.S.: United States
