# Supplementary material for: Increasing nontuberculous mycobacteria reporting rates and species diversity identified in clinical laboratory reports
Source: BMC Infect Dis. 2018 Apr 10;18:163. doi: 10.1186/s12879-018-3043-7 (PMC5891905; doi:10.1186/s12879-018-3043-7)
Supplement: Supplementary file 1 — Table S1. Nontuberculous mycobacteria reported by Missouri, 2014. Table S2. Nontuberculous mycobacteria reported by Mississippi, 2014; Table S3. Nontuberculous mycobacteria reported by Ohio, 2014. Table S4. Nontuberculous mycobacteria reported by Wisconsin, 2014. Provisional counts of NTM reports by State and NTM Species. (DOCX 26 kb) [file 12879_2018_3043_MOESM1_ESM.docx]

Additional file 1

Maura J. Donohue,*^†^

Increasing Nontuberculous Mycobacteria Reporting Rates and Species Diversity Identified in Clinical Laboratory Reports

Number of Pages: 5 (includes coversheet)

Supplementary Table S1: Nontuberculous mycobacteria reported by Missouri, 2014.

Supplementary Table S2: Nontuberculous mycobacteria reported by Mississippi, 2014

Supplementary Table S3: Nontuberculous mycobacteria reported by Ohio, 2014.

Supplementary Table S4: Nontuberculous mycobacteria reported by Wisconsin, 2014.

Supplementary Table S1: Nontuberculous mycobacteria reported by Missouri, 2014.

| State |  | Complex | M. Species | Number of Reports |
| --- | --- | --- | --- | --- |
| MO |  | M. terrae Complex | *M. arupense* | 18 |
| MO |  | Ungrouped | *M. aurum* | 1 |
| MO |  | MAC | *M. avium* | 394 |
| MO |  | M. chelonae-abscessus Group | *M. bollettii* | 2 |
| MO |  | Ungrouped | *M. branderi* | 1 |
| MO |  | M. chelonae-abscessus Group | *M. chelonae* | 40 |
| MO |  | Ungrouped | *M. cosmeticum* | 3 |
| MO |  | M. fortuitum Group | *M. fortuitum* | 40 |
| MO |  | M. fortuitum Group | *M. fortuitum* complex | 25 |
| MO |  | Ungrouped | *M. frederiksbergense* | 1 |
| MO |  | M. smegmatis Group | *M. goodii* | 1 |
| MO |  | M. gordonae | *M. gordonae* | 152 |
| MO |  | M. haemphilem Group | *M. haemophilum* | 2 |
| MO |  | M. fortuitum Group | *M. houstonense* | 1 |
| MO |  | M. chelonae-abscessus Group | *M. immunogenum* | 4 |
| MO |  | M. simiae complex | *M. interjectum* | 1 |
| MO |  | MAC | *M. intracellulare* | 4 |
| MO |  | M. kansasii | *M. kansasii* | 29 |
| MO |  | M. fortuitum Group | *M. mageritense* | 4 |
| MO |  | M. chelonae-abscessus Group | *M. massiliense* | 1 |
| MO |  | M. mucogenicum -phocaicum | *M. mucogenicum* | 42 |
| MO |  | Ungrouped | *M. neoaurum* | 2 |
| MO |  | M. fortuitum Group | *M. neworleansense* | 1 |
| MO |  | M. terrae Complex | *M. nonchromogenicum* | 2 |
| MO |  | Ungrouped | *M. paraffinicum* | 2 |
| MO |  | M. simiae complex | *M. parascrofulaceum* | 1 |
| MO |  | M. fortuitum Group | *M. peregrinum* | 2 |
| MO |  | M. mucogenicum -phocaicum | *M. phociacum* | 1 |
| MO |  | M. fortuitum Group | *M. porcinum* | 3 |
| MO |  | M. simiae complex | *M. simiae* | 1 |
| MO |  | M. smegmatis Group | *M. smegmatis* | 1 |
| MO |  | Ungrouped | *M. sphagni* | 1 |
| MO |  | Ungrouped | *M. szulgai* | 3 |
| MO |  | M. terrae Complex | *M. terrae* | 13 |
| MO |  | M. simiae Complex | *M. triplex* | 1 |
| MO |  | M. smegmatis Group | *M. wolinskyi* | 1 |
| MO |  | M. xenopi | *M. xenopi* | 6 |
| MO |  | MAIS | MAIS COMPLEX | 2 |
| MO |  | NTM | Mycobacterium Species | 47 |
| MO |  | NTM | Mycobacterium Unknown | 18 |
| MO |  | NTM | OTHER RAPID GROWER | 5 |
| MO |  | **Total** |  | **879** |

MOTT reports equate to the number of unique patients reporting positive specimens and includes the condition statues of clinical case, confirmed, doctor’s diagnosis, lab confirmed and probable.

Missouri Health Department Disclaimer: Report numbers are provisional.

Supplementary Table S2. Nontuberculous mycobacteria reported by Mississippi, 2014

| State |  | Complex | M. Species | Number of Reports |
| --- | --- | --- | --- | --- |
| MS |  | NTM | Mycobacterium spp | 23 |
| MS |  | M. chelonae-abscessus Group | *M. abscessus* | 2 |
| MS |  | M. chelonae-abscessus Group | *M. chelonae* | 30 |
| MS |  | M. fortuitum Group | *M. fortuitum* | 24 |
| MS |  | M. gordonae | *M. gordonae* | 101 |
| MS |  | M. kansasii | *M. kansasii* | 30 |
| MS |  | M. xenopi | *M. xenopi* | 1 |
| MS |  | MAC | *M. avium* | 297 |
| MS |  | MAC | *M. intracellulare* | 1 |
| MS |  | MAIS | *M. scrofulaceum* | 1 |
| MS |  | M. mucogenicum -phocaicum | *M. mucogenicum* | 10 |
| MS |  | Ungrouped | *M. Algericum* | 1 |
| MS |  | M. terrae Complex | *M. nonchromogenicum* | 4 |
| MS |  | M. terrae Complex | *M. terrae* | 2 |
| MS |  | Ungrouped | *M. paraffinicum* | 1 |
| MS |  | Ungrouped | *M. szulgai* | 1 |
| MS |  | **Total** |  | **529** |

Reports equate to the number of unique patients reporting positive specimens.

Mississippi Health Department Disclaimer: Report numbers are provisional.

Supplementary Table S3 Nontuberculous mycobacteria reported by Ohio, 2014

| State |  | Complex | M. Species | Number of Reports |
| --- | --- | --- | --- | --- |
| OH |  | Ungrouped | *M. iranicum* | 1 |
| OH |  | M. chelonae-abscessus Group | *M. abscessus* | 31 |
| OH |  | M. chelonae-abscessus Group | *M. bolletii* | 2 |
| OH |  | MAC | *M. avium* | 14 |
| OH |  | MAC | *M. avium* complex | 620 |
| OH |  | Ungrouped | *M. bacteremicum* | 1 |
| OH |  | M. chelonae-abscessus Group | *M. bolletii* | 1 |
| OH |  | M. chelonae-abscessus Group | *M. chelonae* | 32 |
| OH |  | M. chelonae-abscessus Group | *M. chelonae/abscessus* group | 76 |
| OH |  | M. fortuitum Group | *M. conceptionense* | 2 |
| OH |  | Ungrouped | *M. cosmeticum* | 1 |
| OH |  | M. fortuitum Group | *M. fortuitum* | 46 |
| OH |  | M. fortuitum Group | *M. fortuitum* complex | 53 |
|  |  | M. fortuitum Group | *M. peregrinum* | 51 |
| OH |  | M. kansasii | *M. gastri* | 1 |
| OH |  | M. smegmatis Group | *M. goodii* | 2 |
| OH |  | M. smegmatis Group | *M. goodii* complex | 3 |
| OH |  | M. gordonae | *M. gordonae* | 247 |
| OH |  | M. simiae Complex | *M. interjectum* | 1 |
| OH |  | MAC | *M. intracellulare* | 1 |
| OH |  | M. kansasii Clade | *M. kansasii* | 26 |
| OH |  | M. simiae Complex | *M. kubicae* | 2 |
| OH |  | M. simiae Complex | *M. lentiflavum* | 13 |
| OH |  | M. haemphilem Group | *M. malmoense* | 3 |
| OH |  | M. mucogenicum -phocaicum | *M. mucogenicum* | 30 |
| OH |  | M. mucogenicum -phocaicum | *M. mucogenicum/ phocaicum* | 4 |
| OH |  | Ungrouped | *M. neoaurum* | 3 |
| OH |  | M. fortuitum Group | *M. neworleansense/porcinum* | 1 |
| OH |  | M. terrae Complex | *M. nonchromogenicum* | 4 |
| OH |  | M. terrae Complex | *M. terrae* | 2 |
| OH |  | Ungrouped | *M. paraffinicum* | 5 |
| OH |  | M. simiae Complex | *M. parascrofulaceum* | 1 |
| OH |  | M. fortuitum Group | *M. peregrinum* | 4 |
| OH |  | M. fortuitum Group | *M. peregrinum/septicum* | 4 |
| OH |  | M. fortuitum Group | *M. porcinum* | 1 |
| OH |  | Ungroup | *M. pulveris* | 1 |
| OH |  | MAIS | *M. scrofulaceum* | 1 |
| OH |  | M. fortuitum Group | *M. septicum* | 1 |
| OH |  | M. simiae complex | *M. simiae* | 19 |
| OH |  | M. smegmatis Group | *M. smegmatis* | 2 |
| OH |  | Ungrouped | *M. szulgai* | 3 |
| OH |  | M. smegmatis Group | *M. wolinskyi* | 1 |
| OH |  | M. xenopi | *M. xenopi* | 10 |
| OH |  | MAIS | MAIS COMPLEX | 15 |
| OH |  | NTM | Mycobacterium Non-tuberculous mycobacterium (not identified) | 37 |
| OH |  | **Total** |  | **1379** |

Reports equate to the number of unique patients reporting positive specimens.

Ohio Health Department Disclaimer: Report case numbers are underestimated: 1. Some reports case may contain more than one organism, and 2. Some patients may have two or more lab results in a case that should have been reported as separate cases.

Supplementary Table S4. Nontuberculous mycobacteria reported by Wisconsin, 2014

| State |  | Complex | M. Species | Number of Reports |
| --- | --- | --- | --- | --- |
| WI |  | M. chelonae-abscessus Group | *M. abscessus* | 29 |
| WI |  | M. terrae Complex | *M. arupense* | 3 |
| WI |  | MAC | *M. avium* complex | 921 |
| WI |  | Ungrouped | *M. celatum* | 1 |
| WI |  | M. chelonae-abscessus Group | *M. chelonae* | 33 |
| WI |  | M. chelonae-abscessus Group | M. chelonae-abscessus group | 46 |
| WI |  | Ungrouped | *M. conceptionense* | 1 |
| WI |  | M. fortuitum Group | *M. fortuitum* group | 65 |
| WI |  | M. chelonae-abscessus Group | *M. franklinii* | 1 |
| WI |  | M. smegmatis Group | *M. goodii* | 1 |
| WI |  | M. gordonae | *M. gordonae* | 188 |
| WI |  | M. kansasii | *M. kansasii* | 14 |
| WI |  | M. simiae complex | *M. kubicae* | 1 |
| WI |  | M. terrae Complex | *M. kumamotonense* | 1 |
| WI |  | M. simiae complex | *M. lentiflavum* | 2 |
| WI |  | M. haemphilem Group | *M. malmoense* | 2 |
| WI |  | M. chelonae-abscessus Group | *M. massiliense* | 1 |
| WI |  | Mucogenicum Clade | *M. mucogenicum* | 22 |
| WI |  | Ungrouped | *M. nebraskense* | 2 |
| WI |  | Ungrouped | *M. neoaurum* | 1 |
| WI |  | Ungrouped | *M. paraffinicum* | 1 |
| WI |  | M. fortuitum Group | *M. peregrinum* | 15 |
| WI |  | M. fortuitum Group | *M. porcinum* | 1 |
| WI |  | MAIS | *M. scrofulaceum* | 1 |
| WI |  | M. fortuitum Group | *M. septicum* | 3 |
| WI |  | M. simiae complex | *M. simiae* complex | 2 |
| WI |  | M. smegmatis Group | *M. smegmatis* group | 1 |
| WI |  | Ungrouped | *M. szulgai* | 3 |
| WI |  | M. terrae Complex | *M. terrae complex* | 11 |
| WI |  | Ungrouped | *M. vaccae* | 1 |
| WI |  | M. xenopi | *M. xenopi* | 37 |
| WI |  | NTM | NTM | 2 |
| WI |  | **Total** |  | **1413** |

Reports equate to number of patients with a new isolates of *Mycobacterium* species, (non-tuberculous)

Wisconsin’s Health Department Disclaimer: Report numbers are provisional.
